# Assessment of Salivary Parameters—pH, Buffering Capacity and Flow-Associated with Caries Susceptibility

**DOI:** 10.3390/diagnostics16040625

**Published:** 2026-02-20

**Authors:** Alexandru Ștefârță, Mihaela Roxana Brătoiu, Maria Alexandra Rădoi, Veronica Mercuț, Mihaela Ionescu, Monica Scrieciu, Ileana-Cristiana Petcu, Petre-Costin Mărășescu, Marina Olimpia Amărăscu, Adrian Marcel Popescu, Diana-Elena Vlăduțu

**Affiliations:** 1 Department of Prosthetic Dentistry, University of Medicine and Pharmacy of Craiova, 200349 Craiova, Romania; alexandru.stefarta@umfcv.ro (A.Ș.); veronica.mercut@umfcv.ro (V.M.); monica.scrieciu@umfcv.ro (M.S.); cristiana.petcu@umfcv.ro (I.-C.P.); smpopescu@mail.com (A.M.P.); diana.vladutu@umfcv.ro (D.-E.V.); 2 Department of Medical Informatics and Biostatistics, University of Medicine and Pharmacy of Craiova, 200349 Craiova, Romania; 3 Department of Dental Prosthesis Technology, University of Medicine and Pharmacy of Craiova, 200349 Craiova, Romania; marasescup@yahoo.com; 4 Department of Dental Morphology, University of Medicine and Pharmacy of Craiova, 200349 Craiova, Romania; marina.amarascu@umfcv.ro

**Keywords:** caries susceptibility, DMFT index, salivary pH, buffering capacity, flow rate

## Abstract

**Background/Objectives:** Saliva plays an essential role in maintaining the oral ecological balance, and its quantitative and qualitative characteristics may influence susceptibility to dental caries. The aim of this study was to determine susceptibility to dental caries based on the DMFT index and to establish a correlation between caries experience and salivary parameters in a group of young adults. **Methods:** This cross-sectional study was conducted between July and November 2025 on a sample of 87 fourth-year students from the Faculty of Dentistry in Craiova. Each participant underwent an intraoral clinical examination to determine the DMFT index. The salivary parameters assessed included unstimulated salivary flow rate, saliva consistency, salivary pH, stimulated salivary flow rate, and buffering capacity, using the GC Saliva-Check Buffer kit. Statistical analyses were performed using SPSS (Statistical Package for Social Sciences) software, version 26 (SPSS Inc., Armonk, NY, USA). **Results**: The mean DMFT index value for the entire sample was 8.26 ± 4.481, with higher values observed among female participants. Low salivary pH was significantly associated with higher DMFT values. Participants with low or very low buffering capacity exhibited higher DMFT values compared to those with normal capacity, indicating that a reduced ability to neutralize salivary acidity is associated with increased caries activity. **Conclusions:** The results indicate that salivary pH and buffering capacity are important factors in dental caries susceptibility among young adults. The integration of salivary testing into the diagnostic assessment of caries risk may contribute to personalized and effective preventive strategies.

## 1. Introduction

Dental caries is a highly prevalent non-communicable disease, affecting people of all ages and having a significant impact on public health according to the Global Oral Health Status Report published by the World Health Organization (WHO) in 2022 [1]. The term dental caries refers both to the condition and to the damage caused to the teeth. Dental caries is considered a foodborne microbial disease, requiring a cariogenic biofilm and regular exposure to fermentable carbohydrates in the diet (glucose, fructose, maltose, and sucrose) [2,3,4].

Dental caries is a major health problem in most developed countries, with an estimated prevalence of 46.2% in the primary dentition and 53.8% in the permanent dentition among children [5,6].

According to the “GBD 2017 Oral Disorders Collaborators”, globally, in 2017, 3.5 billion cases of oral diseases were recorded, of which 2.3 billion were represented by untreated caries in permanent teeth, 532 million were represented by untreated caries in primary teeth and 267 million cases were completely edentulous [7], and in 2019, 3.09 billion new cases of untreated dental caries in permanent teeth were reported [8]. Contrary to previous data, the peak prevalence of untreated dental caries in the permanent dentition was observed in the younger age group (15–19 years) in 2015 [9,10].

Epidemiological evidence has shown that the prevalence of dental caries has decreased over the past four decades, particularly in high-income countries, with the most substantial decline observed in 12-year-old children [9,11,12]. A 2023 meta-analysis review reported a higher prevalence of dental caries in racially marginalized children, highlighting greater inequalities in dental caries within high-income countries [13].

The index traditionally used in epidemiological studies to measure individual caries experience is the DMFT index (decayed, missing, and filled teeth). The DMFT index records the caries experience of an individual or a group of individuals (i.e., the total of current and past caries). It can be used to determine the degree of dentition damage based on the mean DMFT and the proportion of affected individuals (prevalence) [3,14,15]. This index is also employed to evaluate and monitor the outcomes of oral health interventions in the community, informing the development of related policies and programmes [14,16,17].

The dynamics of carious lesions depend on factors related to the host organism, the availability of fermentable sugars, the oral microbiome, and other conditions of the oral environment [18]. Among the specific conditions of the oral environment that influence the occurrence and progression of caries, tooth mineralization, saliva composition, and oral hygiene should be mentioned [19]. Saliva represents the most important component of the oral environment and is an integral part of oral health [20]. It is an aqueous substance produced by the salivary glands of the oral cavity, composed of approximately 98% water, with the remaining 2% consisting of electrolytes, glycoproteins, and antibacterial components such as immunoglobulins and enzymes, including lysozyme [21,22]. In addition to the secretions of the salivary glands, saliva also contains other components originating from the oropharynx, upper respiratory tract, gastrointestinal reflux, gingival sulcus fluid, food residues, and blood-derived compounds [23,24]. In a healthy adult, approximately 1–1.5 L of saliva is secreted per day, and this amount is distributed over an area of roughly 200 cm^2^ in the oral cavity, forming a thin film with a thickness of approximately 10–100 µm [21,25]. The role of saliva in caries prevention is achieved by maintaining an ecological balance through mechanical, immunological, and non-immunological means, including self-cleaning via lavage and debridement [26].

Saliva is effective in maintaining a relatively neutral pH (potential of hydrogen) in both the oral cavity and dental plaque. At the level of dental plaque, acid production results from carbohydrate metabolism under bacterial activity, while saliva contributes to pH regulation through bicarbonate, phosphate, and histidine-rich peptides, which act directly as buffering agents once diffused into the plaque [20]. Saliva also inhibits bacterial attachment through the action of lysozyme, lactoferrin, and lactoperoxidase, thereby preventing bacterial proliferation [20]. A salivary pH between 6.8 and 7.4 is considered optimal for protecting teeth from acidity produced by the activity of oral microorganisms and dietary intake, which represents the main cause of dental caries [27].

The objectives of this research were to determine the susceptibility to dental caries based on the DMFT index in a group of young students and to establish a correlation between caries susceptibility and salivary parameters, including unstimulated salivary flow rate, saliva consistency, salivary pH, stimulated salivary flow rate, and buffering capacity. The null hypothesis asserts that there is no statistically significant relationship between the DMFT index and salivary parameters.

## 2. Materials and Methods

### 2.1. Study Design

The present study was conducted between July and November 2025 on a group of 87 fourth-year students at the Faculty of Dentistry, Craiova. The sample size was computed using G*Power 3.1.9.7, Heinrich Heine University Düsseldorf, Germany, considering a significance level α of 0.05, a power 1 − β equal to 0.95, and a medium effect size value of 0.4 (with an awareness of practical significance), resulting in a study requirement of a minimum of 82 participants. The study was approved by the University Ethics and Deontology Committee of the University of Medicine and Pharmacy of Craiova no. 242/20.06.2025 and was carried out in compliance with the Declaration of Helsinki [28,29]. After obtaining informed consent from all study participants, an intra-oral clinical examination was performed and the saliva properties associated with salivary pH were determined.

### 2.2. Subject Selection

The *inclusion criteria* for the study were as follows: fourth-year students at the Faculty of Dentistry, Craiova, of both genders; not undergoing treatment with diuretics, antidepressants, antihistamines, antipsychotics, antihypertensives, or aspirin; not having used mouthwash in the morning; not consuming carbonated or energy drinks, herbal or fruit teas; and having provided informed consent to participate in the study.

*The exclusion criteria* were as follows: students who did not provide consent to participate; those who did not comply with preliminary recommendations necessary for accurate measurements; and those absent for any reason on the days of clinical examination.

### 2.3. Examination of the Participants

**Figure 1:**
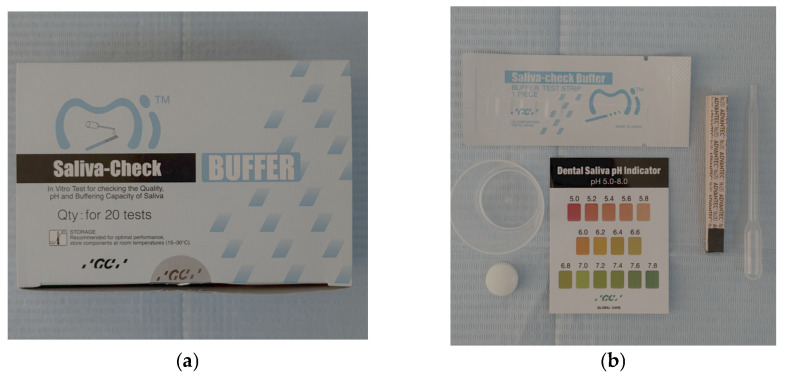
(**a**) GC Saliva check buffer kit. (**b**) In vitro pH strips, saliva collection cup, wax gum for saliva stimulation, saliva dispensing pipette, buffer test strip, direction for use.

For this study, an intra-oral clinical examination was performed to determine the DMFT index, salivary flow, and saliva consistency. Subsequently, salivary pH, salivary buffer capacity, and the amount of stimulated saliva were determined using the GC Saliva-Check Buffer kit (GC, Tokyo, Japan) (Figure 1). All determinations and examinations for a subject were performed in the same session. The intra-oral examination was performed using standard inspection, palpation, and probing techniques in accordance with the World Health Organization and the Declaration of Helsinki [28].

### 2.4. Saliva Assessment

#### 2.4.1. DMFT Index Determination

Each permanent tooth was examined for the presence of caries, fillings, or missing teeth. The DMFT index was calculated as the sum of decayed, missing, and filled teeth.

#### 2.4.2. Salivary Flow

Salivary flow was assessed visually according to the instructions provided in the GC Saliva-Check Buffer kit. The lower lip was everted, and the lower labial vestibular area was gently swabbed with a sterile cotton swab to remove existing saliva. Salivary flow was then evaluated based on the time taken for saliva droplets to appear on the mucosa. Flow was considered normal if droplets appeared in less than 60 s, and low if the appearance took longer than 60 s.

#### 2.4.3. Determination of Saliva Consistency

Salivary consistency was assessed visually. Saliva was considered highly viscous if it had a foamy-sticky appearance, of low viscosity if it appeared foamy and aerated, and of normal consistency if it was clear and watery.

#### 2.4.4. Determination of Salivary pH

Before salivary pH measurement, participants were instructed not to smoke, consume food or beverages, brush their teeth, or use mouthwash for at least one hour. The GC Saliva-Check Buffer kit includes 20 in vitro pH test strips, 20 saliva collection containers, 20 saliva pipettes, 20 buffer capacity test strips, 20 wax pieces, and a test chart for determining pH and buffering capacity. Participants were asked to expectorate saliva into the kit container. A pH test strip was then immersed in the saliva for 10 s, after which the pH was visually assessed by comparing the resulting colour to the kit chart. pH values between 5.0 and 5.8 were classified as hyperacidic, values between 6.0 and 6.6 as moderately acidic, and values between 6.8 and 7.8 as normal.

#### 2.4.5. Stimulated Salivary Flow Assessment

Participants were instructed to chew a piece of wax. After 30 s, saliva collection began in a graduated container, with participants continuing to chew the wax for 5 min, collecting saliva at regular intervals. Salivary volume was classified as very low (<3.5 mL), low (3.5–5.0 mL), and normal (>5.0 mL). Stimulated salivary flow was considered normal if the flow rate ranged between 1.0 and 1.6 mL/min.

#### 2.4.6. Assessment of Salivary Buffering Capacity

Each test strip in the kit for assessing salivary buffering capacity is disposable and individually packaged. One drop of saliva was placed on each pad of the strip using the pipette provided in the kit. The strip was then tilted at a 90-degree angle to remove excess saliva, ensuring the accuracy of the test. The colour change occurs immediately, and the final result was recorded after 2 min, with buffering capacity values assigned according to the colour: green = 4 points, green/blue = 3 points, blue = 2 points, red/blue = 1 point, and red = 0 points. The overall score was calculated by summing the points from all pads. Values of 0–5 were considered very low buffering capacity, 6–9 low, and 10–12 normal. All procedures were performed by a single examiner, and the recorded data were entered into an Excel spreadsheet for statistical analysis. Calibration was performed on a set of 15 randomly selected participants, 30 min later, after the first evaluation. Intra-observer reliability was assessed using the intraclass correlation coefficient (ICC), calculated via a two-way mixed-effects model for absolute agreement, resulting in an excellent agreement (0.934).

### 2.5. Statistical Analysis

Statistical tests were applied using SPSS (Statistical Package for Social Sciences) software, version 26 (SPSS Inc., Armonk, NY, USA). Continuous variables were defined as mean ± standard deviation (SD), as well as medians. Nominal and ordinal variables were described as frequency distributions and percentages. Normality distribution was assessed using the Kolmogorov–Smirnov/Shapiro–Wilk test. Correlations and comparisons between groups were performed with the Mann–Whitney U test for continuous non-normally distributed data, and the Chi-Square test for categorical data. All *p*-values less than 0.05 were considered statistically significant.

## 3. Results

### 3.1. Selection of Study Group

Following the application of the inclusion and exclusion criteria, 87 of the 91 fourth-year students were selected for inclusion in the study.

Of the 87 participants included in the study, 59 were female (67.8%) and 28 were male (32.2%). Their ages ranged from 22 to 30 years, with a mean age of 23.86 years and a standard deviation of 3.82 (Table 1). The study group was homogeneous in terms of age by gender.

### 3.2. Caries Experience Based on DMFT Index

The DMFT value was calculated as the sum of decayed, missing, and filled teeth. The data regarding the DMFT index are presented in Table 2.

Based on the intraoral examination of the participants, the mean DMFT value for the entire study group was 8.26 ± 4.48. Female participants presented a higher mean DMFT value (8.83 ± 4.45), while male participants showed a lower mean value (7.07 ± 4.40).

Regarding the DMFT components, the mean number of decayed teeth was 2.82 ± 2.35 for the whole group, 2.75 ± 2.17 in females, and 2.96 ± 2.72 in males.

The missing teeth index was 0.701 with a standard deviation of 1.231 for the entire study group, with a mean value of 0.780 ± 1.340 among female participants and 0.536 ± 0.962 among male participants.

The filled teeth index was 4.747 with a standard deviation of 3.167 for the whole group, with a mean value of 5.305 ± 3.153 for female participants and, respectively, 3.571 ± 2.911 for male participants.

### 3.3. Assessment of Unstimulated and Stimulated Saliva Parameters

The parameters of unstimulated and stimulated saliva are presented in Table 3.

Regarding salivary flow, the distribution of cases with reduced and normal values was similar between female and male participants (*p* = 0.941), indicating that resting salivary secretion is not influenced by gender.

The consistency of saliva was predominantly “frothy/bubbly” in both genders, followed by “watery/clear” consistency, without significant differences (*p* = 0.369). It should be noted, however, that the “sticky frothy” form occurred exclusively in female participants, although its low frequency did not have a statistically significant effect on the results.

Analysis of salivary pH revealed a significant difference between genders (*p* = 0.046), with median values higher in men (7.00) compared to women (6.60).

In the analysis of pH categories, significant differences were observed (*p* = 0.003). For the statistical test, the two female participants with very low pH were grouped with those with low pH, resulting in no cells with expected frequencies less than 5. Overall, women presented much more frequently very low and low pH values (77.97%), while men predominated in the normal pH category (53.6%). This result indicates the presence of a more acidic pH in women.

The results regarding the amount of stimulated saliva confirmed this trend, with men presenting a significantly higher mean secretion (8.0 mL) compared to women (7.0 mL, *p* = 0.021). These two differences indicate a more active secretory function and potentially better salivary protection among men.

In contrast, the buffer capacity categories (*p* = 0.322) and the stimulated saliva category (with very low and low categories grouped) (*p* = 0.214) did not show significant differences between genders.

### 3.4. DMFT Analysis According to Salivary Parameters

Analysis of the relationship between DMFT index and salivary flow did not reveal statistically significant differences between individuals with low salivary flow and those with normal salivary flow (*p* = 0.643) (Table 4). The median and mean DMFT values were similar in both groups (median = 8.000), suggesting that, within the studied group, decreased salivary flow did not have a significant impact on the caries experience.

Analysis of differences in DMFT values according to saliva consistency did not show a statistically significant association (*p* = 0.323) (Table 5). Although mean DMFT values were slightly lower in participants with “sticky frothy” saliva compared to those with “frothy bubbly” or “watery clear” saliva, the differences were not statistically significant. The results suggest that the type of saliva consistency does not significantly influence the caries experience within the analyzed group.

The results indicate a statistically significant difference between DMFT index values and salivary pH categories (*p* = 0.012) (Table 6). Participants with very low or low salivary pH exhibited higher DMFT index values compared to those with normal pH. These findings suggest that increased salivary acidity is associated with greater caries experience, confirming the protective role of normal salivary pH in maintaining oral health.

A statistically significant difference was observed between DMFT index values and salivary buffering capacity categories (*p* = 0.030) (Table 7). Participants with low or very low buffering capacity exhibited higher DMFT values compared to those with normal capacity, indicating that a reduced ability to neutralize salivary acidity is associated with increased caries activity.

A multiple regression was run with DMFT as the outcome, as well as gender, salivary flow, pH value, and buffering capacity, as predictors. There was linearity, as assessed by partial regression plots and a plot of studentized residuals against the predicted values. There was independence of residuals, and homoscedasticity was assessed by visual inspection of a plot of studentized residuals versus unstandardized predicted values. The assumption of normality was met, as assessed by a Q-Q plot. The multiple regression model statistically significantly predicted the DMFT index, F(4, 82) = 2.693, *p* = 0.037, adjusted R^2^ = 0.073. Only the pH value added statistically significantly to the prediction, *p* = 0.014 (Table 8), while the other predictors did not add significance to the DMFT value.

As such, an increase in the pH value of one unit is associated with a decrease in the DMFT index of 3.553, given the fact that the slope coefficient is negative. Thus, the higher the pH value, the lower the DMFT index, when all other independent variables are held constant.

## 4. Discussion

The aim of this study was to evaluate the relationship between caries experience, expressed by the DMFT index, and salivary parameters (salivary flow, consistency, pH, stimulated saliva volume, and buffering capacity) in a group of 87 students from the Faculty of Dentistry, Craiova. Studies conducted on student populations are quite common for assessing various oral parameters [30,31].

The oral cavity is often described as the ‘window to general health’ due to the interrelationship between oral and overall health [32,33,34]. Although remarkable progress has been made in the prevention and treatment of dental diseases, oral diseases remain a significant burden on society [35]. Numerous studies support the association between poor oral health and systemic conditions such as cardiovascular diseases, diabetes, and chronic obstructive pulmonary disease (COPD) [36,37,38,39].

The DMFT index is the most widely used indicator for assessing oral health [40] and is recognized by the World Health Organization for quantifying and comparing the prevalence of dental caries in populations [41]. In the present study, the DMFT index showed moderate values, with a mean of 8.26, comparable to similar studies conducted on young populations in Europe [42]. Studies involving older age groups report higher DMFT values [43].

Within the DMFT index recorded in the study, filled teeth accounted for the largest proportion, followed by teeth with active caries and missing teeth. Higher DMFT values were observed in female participants, which has been explained in the literature by earlier tooth eruption in girls, easier access to food, frequent snacking during meal preparation, and pregnancy [44]. Additionally, it should be noted that female participants in this study exhibited a more acidic salivary pH. Other studies have reported results comparable to those obtained in the present study [42,45].

Regarding the role of saliva in caries predisposition, a series of salivary parameters related to both unstimulated and stimulated saliva were evaluated in the present study. Salivary components play an important role in maintaining oral health [46]. The carioprotective role of saliva can be considered to involve four mechanisms: dilution and clearance of sugars and other substances, buffering of oral acidity, regulation of demineralization and remineralization, and antimicrobial activity [47].

In the present study, unstimulated salivary flow did not show a significant association with the DMFT index. This result is consistent with some studies showing that, in young and healthy individuals, variations in salivary flow are usually within physiological limits, without producing obvious clinical effects on caries risk [48]. Reduced salivary flow becomes particularly relevant in patients with severe xerostomia, patients with anticholinergic medication, or systemic diseases [49]. Other studies, including that carried out by Aline dos Santos Letieri et al. 2022 [50], have shown that the importance of saliva is evident in individuals with low salivary flow, who present acute irritation of the oral mucosa, dental caries and severe difficulties with speech, swallowing, food elimination and taste [50]. Saliva obviously functions to limit, rather than completely eradicate, colonization of oral tissues by microorganisms. Patients with low salivary flow are more susceptible to dental caries and fungal infections than healthy individuals [24,50].

Constantly washing the teeth and oral mucosa with saliva, it functions as a cleaning solution, lubricant, buffer, and reservoir of calcium and phosphate ions, essential for the remineralization of initial carious lesions [51]. The study did not reveal an association between caries predisposition and unstimulated salivary flow or saliva consistency. Saliva acts as the first line of defence against acids produced by cariogenic bacteria in the oral cavity. By neutralizing these acids, saliva helps prevent demineralization of tooth enamel and promotes remineralization, thus reducing the risk of developing cavities [52].

Saliva consistency, an often underestimated clinical parameter, did not significantly influence DMFT, although the lowest DMFT values were observed in the group with ‘sticky frothy’ saliva. However, this subgroup was very small (*n* = 4), which limits statistical interpretation. In general, viscous saliva is associated in the literature with an increased caries risk, as it reduces the ability of saliva to remove food debris and neutralize acids. The absence of an association in the present study may be explained by the young age and overall good health of the participants. Some studies demonstrate that increased salivary viscosity is a risk factor for dental caries [53]. Moreover, increased salivary viscosity is strongly associated with decreased salivary flow, which itself is a risk factor for dental caries [54].

In the present study, categorical analysis of pH revealed a strong association between low pH and high DMFT values. Saliva composition plays a major role in maintaining and increasing the pH of the biofilm. Among the main constituents that contribute to pH elevation are sialin (containing arginine and lysine) and urea. The subsequent hydrolysis of these molecules releases ammonia, which directly contributes to increasing pH. To maintain oral health and the integrity of dental tissues, salivary pH should be maintained around 6.7, while a low or critical pH is considered to be at or below 5.5. The concentration and activity of ions are responsible for demineralization and remineralization processes through their effect on the solubility of hydroxyapatite [55,56]. In general, lower pH levels increase hard tissue dissolution. When biofilm pH remains below a critical threshold for an extended period, progressive demineralisation occurs, resulting in the loss of calcium and phosphate from the tooth’s mineral structure [57].

The buffering capacity of saliva is more effective in stimulated saliva due to increased bicarbonate secretion and CO_2_ loss, which leads to a rise in pH [58]. Buffering capacity helps neutralize acids produced by acidogenic microorganisms, thereby protecting teeth and preventing enamel demineralization through salivary components such as bicarbonate, phosphate, and protein buffers [46,59].

In the present study, the most important findings were derived from the analysis of salivary pH and buffering capacity, both of which demonstrated significant associations with caries experience. Salivary pH values differed significantly between genders, with men exhibiting a more alkaline pH. This difference may be explained by physiological factors, such as variations in metabolic and hormonal rates, diet, and differently stimulated glandular secretion.

Similarly, buffering capacity, which is essential for neutralizing acids, was significantly correlated with DMFT. Participants with very low or low buffering capacity exhibited significantly higher DMFT values than those with normal buffering capacity. This observation is fully consistent with the etiological model of dental caries, in which saliva with reduced buffering capacity allows acidic pH to persist for extended periods, thereby increasing enamel mineral damage [60].

The amount of stimulated saliva was greater in men, a finding frequently reported in physiological studies, as the major salivary glands in men are slightly larger. Although stimulated salivary flow did not show significant differences in relation to DMFT, it is important to note that stimulated salivation is an indicator of glandular reactivity and plays a crucial role during mealtime, when acid neutralization is essential.

The main factor affecting salivary composition is flow. As flow rate increases, pH and the concentration of several constituents, including bicarbonate, protein, sodium, and chloride, also increase. Bicarbonate increases dramatically in stimulated saliva, acting as an effective buffer system [61,62].

The present study indicates that pH and buffering capacity are the most relevant factors associated with caries experience in this group of young adults. These parameters showed a strong association with DMFT, whereas salivary flow and consistency had a minor impact. This profile suggests that, among healthy young adults, qualitative variations in saliva are more important than quantitative ones. Accordingly, preventive interventions should focus on controlling acidic and sugary diets, increasing the frequency of oral hygiene, using fluoride toothpaste, rinsing with alkalizing agents or products that enhance buffering capacity, and periodically monitoring salivary pH in individuals at high risk of caries.

The limitations of the study include the small sample size (87 participants), the narrow age range (average 23 years), and the high level of oral health education, which may not reflect the general population. Additionally, the cross-sectional design does not allow causal relationships between caries susceptibility and specific salivary parameters to be established, but only associations.

More relevant results could be obtained if the study were replicated on healthy subjects, with average oral health education, but also on subjects undergoing a general treatment that has the adverse effects of reducing salivary flow [63].

## 5. Conclusions

The present study was conducted on a group of young people with good oral health education. The results do not reflect the oral health situation of the general population. However, the present study confirms the major protective role of normal salivary pH and adequate buffering capacity in the prevention of dental caries.

Although salivary flow and consistency were not significantly associated with the DMFT index in the study group, salivary parameters demonstrated a crucial influence on caries susceptibility.

These results support the inclusion of salivary testing in the diagnostic assessment of caries risk and emphasize the need for personalized preventive interventions based on the oral biological characteristics of each patient.

## Figures and Tables

**Table 1:** Study group.

Parameter	Category	Gender			*p* *
		Females	Males	Total	
		59 (67.80%)	28 (32.20%)	87 (100%)	
Age (years old)	Median	22.00	23.00	23.00	0.293
	Mean ± SD	23.95 ± 4.357	23.68 ± 2.389	23.86 ± 3.82	

* Mann–Whitney U test. The values in grey are summed by columns.

**Table 2:** DMFT index.

Gender	F	M	Total	*p* *
DMFT index	8.831 ± 4.446	7.071 ± 4.396	8.26 ± 4.481	0.076
Decayed teeth	2.746 ± 2.17	2.964 ± 2.715	2.816 ± 2.345	0.901
Missing teeth	0.78 ± 1.34	0.536 ± 0.962	0.701 ± 1.231	0.561
Filled teeth	5.305 ± 3.153	3.571 ± 2.911	4.747 ± 3.167	0.025

* Mann–Whitney U test.

**Table 3:** Salivary parameters according to gender.

Parameter	Category	Gender			*p*
		Females	Males	Total	
		59 (67.80%)	28 (32.20%)	87 (100%)	
Salivary flux	Low	30 (68.18%)	14 (31.82%)	44 (100%)	
		50.85%	50%		0.941 **
	Normal	29 (67.44%)	14 (32.56%)	43 (100%)	
		49.15%	50%		
Saliva consistency	Frothy bubbly	31 (65.96%)	16 (34.04%)	47 (100%)	
		52.54%	57.14%		
	Sticky frothy	4 (100%)	0 (0%)	4 (100%)	0.369 **
		6.78%	0%		
	Watery clear	24 (66.67%)	12 (33.33%)	36 (100%)	
		40.68%	42.86%		
pH category	Very low	2 (100%)	0 (0%)	2 (100%)	
		3.39%	0%		
	Low	44 (77.19%)	13 (22.81%)	57 (100%)	0.003 *
		74.58%	46.43%		
	Normal	13 (46.43%)	15 (53.57%)	28 (100%)	
		22.03%	53.57%		
Stimulated saliva category	Very low	6 (85.71%)	1 (14.29%)	7 (100%)	
		10.17%	3.57%		
	Low	4 (80%)	1 (20%)	5 (100%)	0.214 *
		6.78%	3.57%		
	Normal	49 (65.33%)	26 (34.67%)	75 (100%)	
		83.05%	92.86%		
Buffer category	Very low	18 (75%)	6 (25%)	24 (100%)	
		30.51%	21.43%		
	Low	23 (67.65%)	11 (32.35%)	34 (100%)	0.322 *
		38.98%	39.29%		
	Normal	18 (62.07%)	11 (37.93%)	29 (100%)	
		30.51%	39.29%		

* Mann–Whitney U test. ** Chi-Square test. The values in grey are summed by columns, representing the distribution of females and males, according to each parameter described in a line. The column Total contains the distribution by gender for each category of the parameters described in a line.

**Table 4:** DMFT analysis according to salivary flow.

Parameter	Category	Salivary Flux			*p* *
		Low	Normal	Total	
		44 (50.57%)	43 (49.43%)	87 (100%)	
DMFT	Median	8.000	8.000	-	0.643
	Mean ± SD	8.386 ± 4.093	8.14 ± 4.892		

* Mann-Whitney U test. The values in grey are summed by columns.

**Table 5:** Analysis of DMFT according to saliva consistency.

Parameter	Category	Saliva consistency				*p* *
		Frothy Bubbly	Sticky Frothy	Watery Clear	Total	
		47 (54.00%)	4 (4.60%)	36 (41.40%)	87 (100%)	
DMFT	Median	7.000	4.000	8.500	-	0.323
	Mean ± SD	8.085 ± 4.122	5.25 ± 5.56	8.833 ± 4.79		

* Mann-Whitney U test. The values in grey are summed by columns.

**Table 6:** DMFT index analysis according to pH category.

Parameter	Category	pH Category				*p* *
		Very Low	Low	Normal	Total	
		2 (2.30%)	57 (65.5%)	28 (32.2%)	87 (100%)	
DMFT index	Median	13.500	10.000	6.000	-	0.012
	Mean ± SD	13.5 ± 9.192	9.088 ± 4.197	6.214 ± 4.058		

* Mann-Whitney U test. The values in grey are summed by columns.

**Table 7:** DMFT index analysis according to buffer capacity category.

Parameter	Category	Buffer Capacity Category				*p* *
		Very low	Low	Normal	Total	
		24 (27.60%)	34 (39.10%)	29 (33.30%)	87 (100%)	
DMFT index	Median	9.500	10.000	5.000	-	0.030
	Mean ± SD	8.917 ± 3.764	9.324 ± 4.866	6.483 ± 4.137		

* Mann-Whitney U test. The values in grey are summed by columns.

**Table 8:** Logistic regression model for DMFT value, based on gender, salivary flow, pH value and buffering capacity.

Parameter	B ^1^	Std. Error	Sig	CI Interval for B	
				Lower	Upper
Gender	−1.139	1.019	0.267	−3.166	0.887
Salivary flow	−0.215	0.935	0.819	−2.074	1.644
pH value	−3.553	1.413	0.014	−6.363	−0.743
Buffering capacity	−0.048	0.141	0.737	−0.329	0.233

^1^ Slope coefficients in the regression equation. Sig represents the statistical significance of the test.

## Data Availability

The authors declare that the data of this research are available from the corresponding authors upon reasonable request.

## Abbreviations

The following abbreviations are used in this manuscript:

WHO	World Health Organization
pH	Potential of hydrogen
DMFT index	Decayed, missing and filled teeth
COPD	Chronic obstructive pulmonary disease
